# Comparative genomic analysis reveals the adaptive traits of *Ralstonia* spp. in aquatic environments

**DOI:** 10.3389/fmicb.2025.1625651

**Published:** 2025-07-30

**Authors:** Gaopeng Liu, Chengzhi Mao, Qi Li, Da Huo, Tao Li

**Affiliations:** ^1^State Key Laboratory of Freshwater Ecology and Biotechnology, Institute of Hydrobiology, Chinese Academy of Sciences, Wuhan, China; ^2^College of Advanced Agricultural Sciences, University of Chinese Academy of Sciences, Beijing, China

**Keywords:** *Ralstonia*, comparative genomics, antibiotic resistance, pathogenic microorganisms, microbial evolution

## Abstract

*Ralstonia* spp. are highly adaptable bacteria that are widely distributed across diverse environments. Here, we isolated four *Ralstonia pickettii* (*R. pickettii*) genomes from cultures of *Dolichospermum* spp., and using a comparative genomic framework of 228 *Ralstonia* genomes. We performed phylogenetic analyses that grouped them into water, soil, plant, and human-associated clades based on their predominant isolation habitats. Fluorescence *in situ* hybridization revealed minimal physical interactions between *R. pickettii* and cyanobacterial cells, indicating a commensal or independent ecological relationship. Distinct differences in carbohydrate-active enzymes (CAZymes) and secondary metabolite profiles were observed between water and human-associated dominant groups compared to plant-associated dominant groups, highlighting potential niche-specific adaptations. The water-associated dominant groups harbored antibiotic resistance genes, including *CeoB* and *OXA*-type *β*-lactamase genes. These genes are typically linked to human-associated strains, suggesting potential horizontal gene transfer or shared selective pressures, and the gene content of T3SS is reduced. Notably, water-associated dominant groups exhibited a unique pyrimidine degradation pathway, potentially enabling the utilization of exogenous pyrimidines to support survival in nutrient-limited aquatic environments. We propose that the gene content loss of T3SS and the acquisition of specialized metabolic pathways reflect adaptive strategies of *Ralstonia* spp. for thriving in aquatic free-living niches.

## 1 Introduction

*Ralstonia* spp., Gram-negative bacteria in the family Burkholderiaceae, are widely distributed across diverse environments, including water, plants, hospitals, and soil (Ryan and Adley, 2014). While their adaptability to various habitats has been extensively studied, the ecological roles and adaptation mechanisms of *Ralstonia* spp. in natural water bodies remain largely unexplored (Yuan et al., 2024; Silva et al., 2024). Previous studies have suggested that genomic islands (GIs) and horizontal gene transfer (HGT) may contribute to the survival of *R. pickettii* in drinking water systems (Yuan et al., 2024), yet little is known about their strategies for adapting to freshwater ecosystems. Several species of *Ralstonia* are opportunistic pathogens with significant ecological and clinical impacts (Fluit et al., 2021; Ryan et al., 2006). *R. pickettii*, in particular, is known to contaminate hospital water systems, increasing the risk of secondary infections. It can also pass through 0.22 μm filter membranes, potentially causing contamination in sterile drug formulations (Demirdag et al., 2022). Meanwhile, factors such as the type III secretion system (T3SS), exopolysaccharides (EPS), and flagella have been identified as key pathogenic determinants in *R. solanacearum* (Peeters et al., 2013). Natural water bodies are rich in microorganisms, which play a vital role in element cycling and energy flow processes. For instance, marine microalgae, which play a crucial role in the global carbon cycle as primary producers (Liu et al., 2020). However, the ecological adaptation mechanisms of *R. pickettii* in non-clinical aquatic environments remain unclear.

However, the ecological significance of *Ralstonia* extends beyond pathogenicity. Whole-genome studies have revealed that *R. eutropha* possess complete pathways for bioplastic synthesis, while deeper insights have shown that *R. pickettii* harbors a complete pathway for microplastic degradation (Ryan et al., 2007; Pohlmann et al., 2006). Comparative genomics has revealed key metabolic adaptations in prokaryotes. A comparison between pathogenic and non-pathogenic *R. solanacearum* genomes identified horizontal gene transfer and gene loss events (Ailloud et al., 2015). In addition, non-synonymous polymorphisms in type III effectors were found to contribute to differences in host range (Ailloud et al., 2015). These virulence genes evolve at a faster rate than the whole of the genome (Remenant et al., 2010). However, a comprehensive phylogenetic and ecological analysis of the genus *Ralstonia* remains lacking. With over 600 genomes from different habitats now available in public databases, there is an unprecedented opportunity to explore the evolutionary relationships and ecological adaptations of *Ralstonia* species across diverse environments. The ecological functions of microorganisms vary across different habitats. Their adaptability is facilitated by mechanisms like HGT and GIs such as antibiotic resistance genes (ARGs), CAZymes, and the type secretion system (TSS) (Zhao et al., 2023; Bernabeu et al., 2024). Getting more understanding of these variations offers a genomic perspective for elucidating the adaptive traits and ecological roles of microorganisms.

In this study, we investigated a *Dolichospermum* spp. bloom event in a freshwater lake in Wuhan, China, and we performed isolation and cultivation, during which *R. pickettii* was found in significant abundance alongside cyanobacterial cells. We obtained four complete genomes from metagenomic data and retrieved genomic data of *Ralstonia* spp. from various habitats via public databases. These genomes were used for phylogenetic and comparative genomic analyses to uncover the ecological and metabolic adaptations of *R. pickettii* to aquatic environments. Additionally, we explored its potential interactions with *Dolichospermum* and examined how pathogenicity-related traits vary across different habitats.

## 2 Materials and methods

### 2.1 Sampling and identification

On May 17th, 2022, a water sample was collected from a natural lake (N: 30.546166, E: 114.353721) in Wuhan, Hubei Province, China. Algae filaments were separated, washed multiple times with distilled water droplets, and then cultured in CT medium. *Ralstonia* isolates were cultured on R2A medium, and after three successive generations of purification, single colonies were selected. The full-length 16S rRNA gene was then directly amplified from these colonies using 2 × EasyTaq^®^ PCR SuperMix (TransGen Biotech, China) with primers 27F (5’-AGAGTTTGATCCTGGCTCAG-3′) and 1492R (5’-TACGGCTACCTTGTTACGACTT-3′). Bacterial identification was conducted using the online BLAST program.^1^ The ncbi-genome-download (v 0.3.3)^2^ tool was employed to download all genome sequences of the *Ralstonia* genus from the RefSeq section, and pyani (v 0.2.12) (Pritchard et al., 2016) software was used to calculate the average nucleotide identity (ANI) for further species identification under -m ANIm -g parameter. The culture used for sequencing was maintained at 28 ± 1°C with a speed of 100 rpm in the incubator. The genomic collection information was compiled based on submission data from NCBI, combined with statistical information from published articles.

### 2.2 Genome sequencing, assembly, and annotation

Metagenomic DNA samples obtained from culture were extracted using the DNA Gel Extraction kit according to the manufacturer’s instructions (Axygen, United States). DNA concentration was measured using a Nanodrop 2000 spectrophotometer (Thermo Scientific, United States). Library construction and sequencing were conducted at BENAGEN Biotechnology Co., Ltd. Sequencing was performed using the Illumina MI seq PE150 platform for next-generation sequencing and the Nanopore PromethION platform for third-generation sequencing, generating 5 Gb of data, respectively. Offline data processing was performed using the fast Guppy basecaller (v 6.3.8) software for accurate base calling with a high-accuracy model and subsequent quality control. FastQC (v 0.11.9) (Schmieder and Edwards, 2011) was used for quality control, and raw data with a quality score below Q20 was discarded. Filtering sequences shorter than 5,000 bp, *de novo* assembly was conducted using the Flye (v 2.9.1)—Racon (v 1.5.0) model (Vaser et al., 2017; Kolmogorov et al., 2019) with nanopore sequences under default parameter, followed by three rounds of correction and polishing. Next, Illumina sequencing data was used for two rounds of polishing of the de novo genome using NextPolish (v 1.4.1) (Hu et al., 2020) software to eliminate sequencing errors under default parameter. *R. pickettii* genomes data were extracted from the complete metagenomic dataset and annotated using Prokka (v 1.14.6) software under default parameter (Seemann, 2014). Full-length 16S rRNA sequences were extracted from the genome using barrnap (v 0.9)^3^ software and identified using the online BLAST program.^4^

### 2.3 Phylogenetic analysis

For comparative analysis, we retrieved 428 *Ralstonia* spp. genome sequences and their annotations from the NCBI GenBank database. We selected 228 genomes with completeness above 99% (evaluated using CheckM (v 1.2.1) (Parks et al., 2015) software under lineage_wf mode) and fewer than 100 contigs for further analysis. Accession numbers of the genomes included in this study and their genomic features are shown in Supplementary Table S1. Protein sequences were extracted from GenBank files using a custom python script, and single-copy orthologous gene families were identified for the 231 genomes (including three species selected as outgroups) using OrthoFinder (v 2.5.5) (Emms and Kelly, 2019) software under -a 50 -M msa parameters. We identified 421 single-copy orthologous proteins with an average length of more than 100 bp for further analysis. After alignment and trimming using MAFFT (v 7.520) (Katoh et al., 2002) software under --auto parameters and TrimAl (v 1.4) software under -automated1 parameters (Capella-Gutiérrez et al., 2009), respectively, conserved proteins for each species were concatenated using SeqKit (v 2.5.1) (Shen et al., 2016) software. The concatenated amino acid sequence of the 421 gene families was used to construct a maximum likelihood (ML) phylogenomic tree with ultrafast 1,000 bootstrap replicates using iqtree2 (v 2.2.5) software under -m MFP -B 1000 -bnni parameters (Nguyen et al., 2015). The amino acid substitution model was automatically selected the best-fit model suggest by iqtree2 software. Phylogenetic trees were visualized and beautified using iTOL (v 5.0) (Letunic and Bork, 2021).

### 2.4 Fluorescence *in situ* hybridization and microscopy

The cultures in logarithmic growth phase were centrifuged at 8000 rpm for 10 min, washed three times with sterile PBS (phosphate buffered saline), and fixed overnight at 4°C in 4% paraformaldehyde. Glass slides were pretreated with 0.1% polylysine (Sangon Biotech, China), onto which the fixed cultures were added and dried at 45°C for more than 20 min to allow adsorption onto the slides. After fixation, pre-hybridization was performed using a hybridization solution and incubated at 40°C for 1 h. Subsequently, a 30 μL solution of probe (5‘-GCAAGGCCTCATGCTATAG-3’, diluted 1,5 v:v in 30% formamide) was added and incubated overnight at 40°C in a moist chamber. Washing steps included 15 min washes with 2 × SSC (Saline sodium citrate), followed by two 7 min washes with 1 × SSC, 15 min wash with 0.5 × SSC, and 45 min incubation with diluted branch probe at 40°C in a moist chamber, followed by 7 min washes with 2 × SSC, 1 × SSC, 0.5 × SSC, and 0.1 × SSC. Incubation at 42°C for 3 h with a fluorescent signal probe in a moist chamber was followed by the same washing process. DAPI solution (2 μg/mL) was incubated for 20 min away from light, and the samples were gently rinsed three times with sterile PBS before sealing the slides with an anti-fluorescence quenching agent. The dilution ratio of SSC solution is expressed as volume ratio (v/v). All reagents were obtained from Wuhan Servicebio Biotechnology Co., Ltd. The design and synthesis of *R. pickettii*’s fluorescent probe were completed by Wuhan Servicebio Biotechnology Co., Ltd. Observations and panoramic scanning were conducted using a Leica microscope Aperio VERSA 8 (v 1.4.0.125). DAPI staining was observed using ultraviolet excitation luminescence, *Dolichospermum* sp. self-luminescence was observed using an excitation wavelength of 510–560 nm (G-2A), and *R. pickettii* was observed emitting green fluorescence in the wavelength range of 460–500 nm.

### 2.5 Comparative genomics analysis

Gene function and secretion system prediction were conducted using KofamScan (v 1.3.0) (Aramaki et al., 2020) software with database version 20,231,127 under default parameter, and the software recommended annotation was selected as the final result. CAZymes annotation was performed using dbCAN2 (v 4.0.0) (Zhang et al., 2018) against the dbCAN2 database (v12) with hmmer-based comparison (coverage >0.35 and e-value <1e-15), dbCAN-sub (coverage >0.35 and e-value <1e-15), and diamond (e-value <1e-10) methods. The annotation predicted by hmmer was used as the statistical standard after integrating the results from three methods. Antibiotic resistance genes (ARGs) and virulence factors were predicted using abricate (v 1.0.1) software^5^ with the Card (Zankari et al., 2012) and VFDB (Chen et al., 2005) databases, respectively (identity >80%). Bacterial secretion systems prediction was extracted from KofamScan software annotated results, and gene names and classification information can be queried through KO numbers. Annotate secondary metabolites using online^6^ and detection strictness used strict, and other parameters using default methods (Blin et al., 2023).

A python script was employed to screen OGs (orthogroups genes) present in all strains, based on the OrthoFinder results excluding outgroups, shared metabolism representing extremely conservative sequences that present in all genomes. One transporter was classified as incomplete because one of its constituent proteins was missing. Nevertheless, this protein showed over 50% identity to the corresponding *Escherichia coli* protein in BLASTp analysis, indicating it may have been overlooked during the prediction process. Such cases are defined as “dismissed” transporter proteins. To investigate the unique metabolic content of each group, an exact Fisher’s test (*p* < 0.05) was performed. This analysis identified whether certain metabolic pathways were specifically lost in individual groups, thereby revealing their unique metabolic profiles. The longest aligned sequence representing each selected OG was chosen, and all sequences were merged for annotation using KofamScan (v 1.3.0) (Aramaki et al., 2020) software with database version 20,231,127. The annotation table was reconstructed using KEGG-reconstruct^7^ to visualize metabolic pathways. A simplified metabolic diagram was created using Adobe Illustrator CC 2019 based on the metabolic pathway results. Gene family contraction and expansion in the *Ralstonia* genus were analyzed using Badirate (v 1.35) (Librado et al., 2012) software based on the BDI-CSP-FR model, providing important insights into species evolution. A standard binary phylogenetic tree, with duplicate values removed, was used for the analysis of gene family contraction and expansion.

### 2.6 Statistical analysis and data visualization

Data were analyzed and visualized using R (v 4.3.1) in RStudio (v 2023.6.0.421) with the R packages pheatmap (v 1.0.12) and ggplot2 (v 2.3.4). Results for CAZymes, resistance genes, and virulence factors were generated using pheatmap (v 1.0.12) and gplots (v 4.3.1). Boxplots for genome-related information were created using ggplot2. The vegan package (v 2.6.4) was utilized for analysis of similarities (anosim) to distinguish among different clades based on CAZymes and secondary metabolites.

## 3 Results

### 3.1 Localize *Ralstonia pickettii* in *Dolichospermum* culture

**Figure 1:**
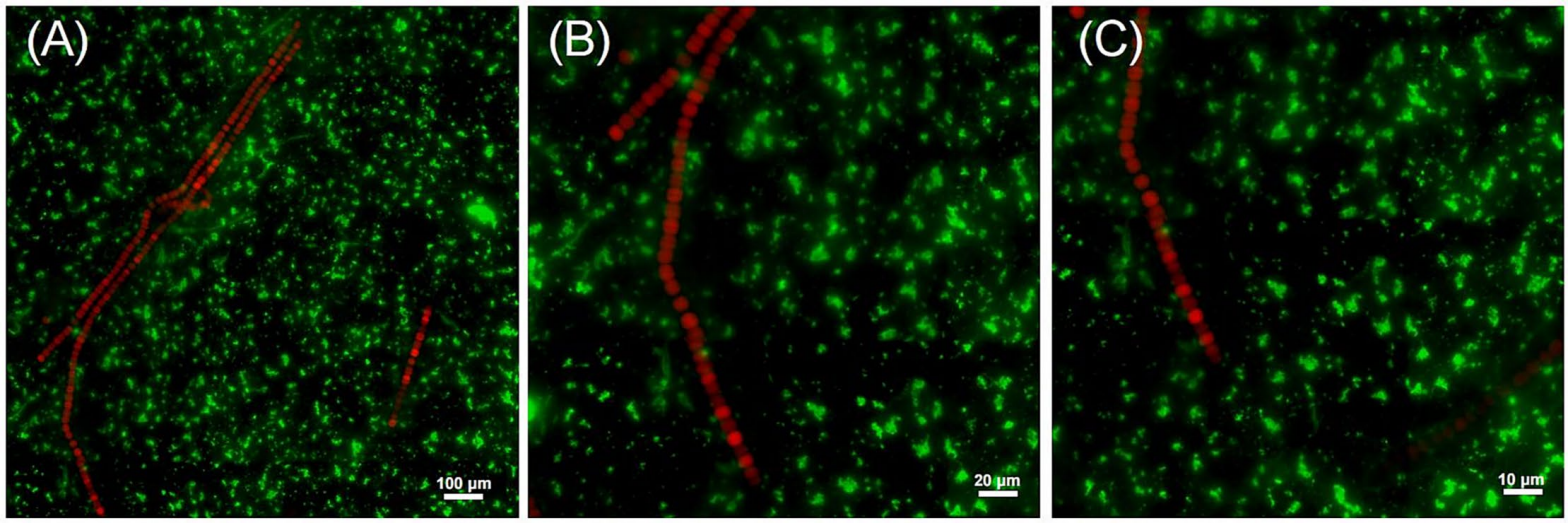
The relative positional relationship between *R. pickettii* and *Dolichospermum* sp. in the culture medium. Using a probe designed based on the 16 s rRNA sequence of *R. pickettii*, light is activated within the 460–500 nm wavelength range (green), whereas the self-luminescence of blue-green algae (red) is activated within the 510–560 nm (G-2A) wavelength range. Maximum intensity projection of stack fluorescence images acquired at magnifications of ×10 **(A)**, ×20 **(B)**, and ×100 **(C)** is shown.

Fluorescence *in situ* hybridization (FISH) was conducted to investigate the spatial relationship between *Dolichospermum* sp. and *R. pickettii* in culture. Individual filaments were isolated under a microscope and washed with sterile water before being transferred to CT medium, a commonly used culture medium for cyanobacteria. During the exponential growth phase of *Dolichospermum* sp., appropriate amounts of culture were collected under sterile conditions for further analysis. The morphology of *Dolichospermum* filaments is distinct, emitting red excitation light *R. pickettii* rarely attaches on the *Dolichospermum* cells (Figures 1A–C). However, there is a small amount of aggregation of *R. pickettii* surrounding the filaments. The abundance of green fluorescent signals from *R. pickettii* in the field of view indicates its high presence in the culture. However, the absence of a stable parasitic relationship suggests that *R. pickettii* is likely to exist in a free-living association with *Dolichospermum*, either in culture or in the natural environment.

### 3.2 Phylogenetic reconstruction

**Figure 2:**
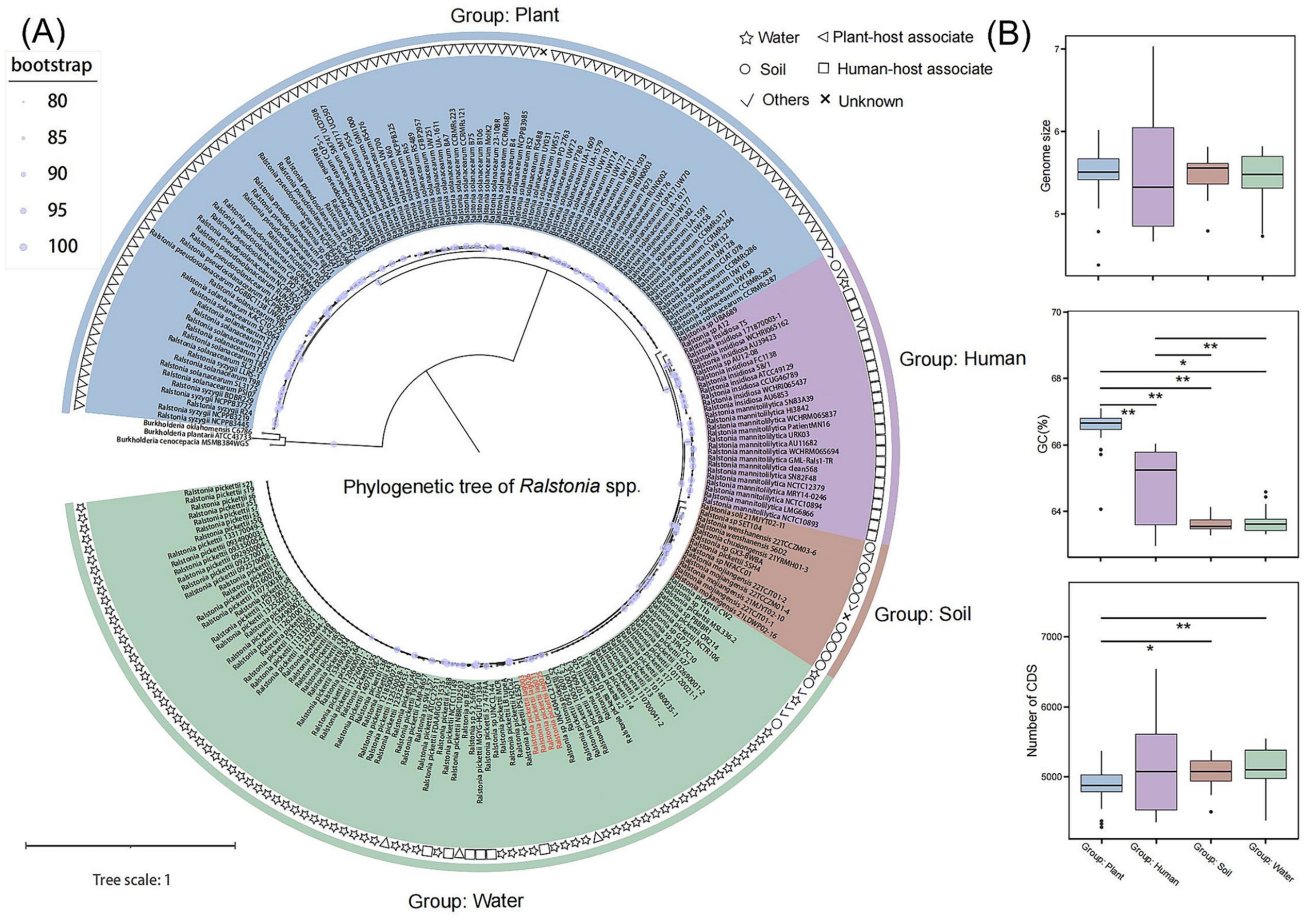
Genomic features and phylogenetic reconstruction of *Ralstonia* spp. genomes. **(A)** Maximum likelihood phylogenetic tree constructed from a concatenated alignment of 421 single-copy orthologous proteins. Nodes with bootstrap values ≥50 are indicated by solid circles. **(B)** Distributions of genome size, GC content (%), and number of coding sequences (CDSs) across different clades. Statistical significance was determined using the Wilcoxon rank-sum test: *p* < 0.05 (*), *p* < 0.01 (**).

We selected 228 genomes with completeness above 99% and fewer than 100 contigs for further analysis. The genomic collection information was compiled based on submission data from NCBI, combined with statistical information from published articles. The ANI results of the 228 genomes (excluding 3 outgroups) (Supplementary Figure S1) indicate that the similarity between the four *Ralstonia* strains which we isolate from *Dolichospermum* sp. culture and *R. pickettii* is over 98%. Based on the primary habitat of the strains within each clade, the 228 species can be categorized into four groups: plant, human, soil and water (Figure 2A). In the plant group, there are *R. solanacearum*, *R. pseudosolanacearum* and *R. syzygii*. There are *R. insidiosa* and *R. mannitolilytica* in the human group, novel species are found in the soil group and *R. pickettii* in the water group. The average genome length and GC content of these 228 *Ralstonia* spp. genomes were 5.49 Mb and 64.99%, ranging from 4.38–7.02 Mb and 62.95–67.1%, respectively (Figure 2B). The greatest variation is the GC content across different habitats, clades associated with plant-hosts comprising the highest proportion, averaging 66.61%, while soil group exhibits the lowest average content at 63.60%. However, the number of CDSs (coding sequences) showed a significant increase in soil and water habitats compared with the plant group (Figure 2B). In this study, it was observed that the number of gene families in the common ancestor of the *Ralstonia* genus was relatively modest, with an increase in gene family count occurring concomitant with adaptation to diverse environments. The *R. insidiosa* gene family, which is associated with human, boasts the highest number of genes and may be implicated in its adaptation to various hosts or environment (Supplementary Figure S2).

### 3.3 Type of toxicity and infectivity in *Ralstonia* spp.

**Figure 3:**
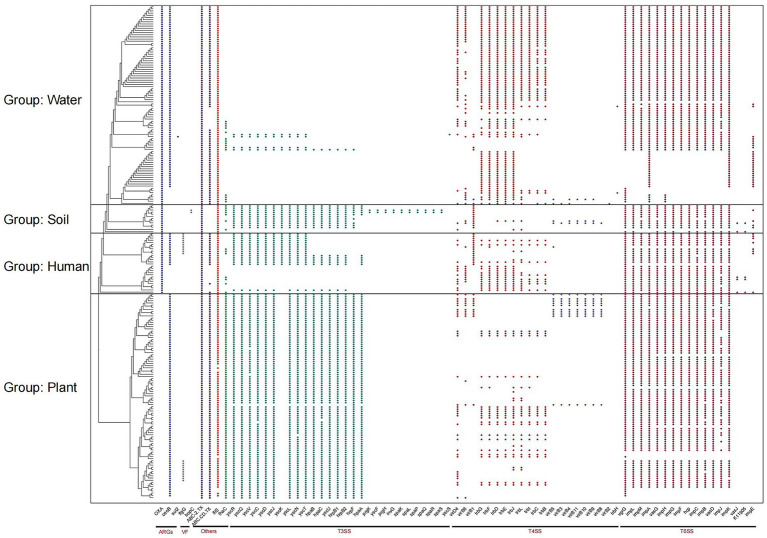
Composition of antibiotic resistance genes (ARGs) and bacterial secretion systems in *Ralstonia* spp. genomes. The figure shows the distribution of ARGs, virulence factors (VFs), type III secretion systems (T3SS), type IV secretion systems (T4SS), type VI secretion systems (T6SS), and other secretion systems in *Ralstonia* spp. genomes.

ARGs are present in nearly all genomes and can be divided into three types: *OXA*, *ceoB*, and *sul2* (Figure 3). *OXA* refers to a class of *β*-lactamases that confer resistance to oxacillin and other β-lactam antibiotics. *CeoB* encodes an efflux pump component involved in resistance to chloramphenicol and other antimicrobials. *Sul2* encodes a variant of dihydropteroate synthase, which mediates resistance to sulfonamide antibiotics. *OXA* is present in all environments except those associated with plant groups. *CeoB* is found in nearly all genomes except those from soil environments, with a notable absence observed in one human-associated lineage. The virulence factor *bopC* is exclusively distributed in soil group, inducing host cell necrosis. *FlgG*, a common virulence factor activating the host’s innate immunity, is found in some groups of plant and human genomes. In water-associated clades, all strains were found to harbor *OXA*- and *ceoB*-type ARGs, indicating a potentially greater threat to host organisms. These findings highlight the importance of monitoring ARGs in *R. pickettii* to minimize the risk of infection associated with environmental exposure or water usage. These findings indicate that *Ralstonia* spp. exhibit distinct patterns of ARGs and virulence factor accumulation across different environments, with aquatic groups showing greater abundance, potentially reflecting the complexity of their ecological niches. Notably, two specific ARGs were identified in aquatic-associated groups and were present in nearly every genome within these clades.

The bacterial secretion system plays a crucial role in the invasion of pathogenic microorganisms into hosts and serves as an indicator of toxicity. All genomes in this study contain complete T2SS, the typical T2SS is a secretion system that transports substances from the periplasmic space to the outer membrane of bacteria, encoded by approximately 12 to 15 *gsp* (general secretion pathway) genes, indicating a potential infection ability in *Ralstonia* spp. T3Es secreted by T3SS play a pivotal role in pathogenesis. Interestingly, the number of T3SS proteins is significantly lower in water group compared to other groups. While T4SS proteins is present in all clades, but with a higher presence observed in water group. Conversely, T6SS exhibits a certain degree of deficiency in water group.

### 3.4 CAZymes in *Ralstonia* genus

**Figure 4:**
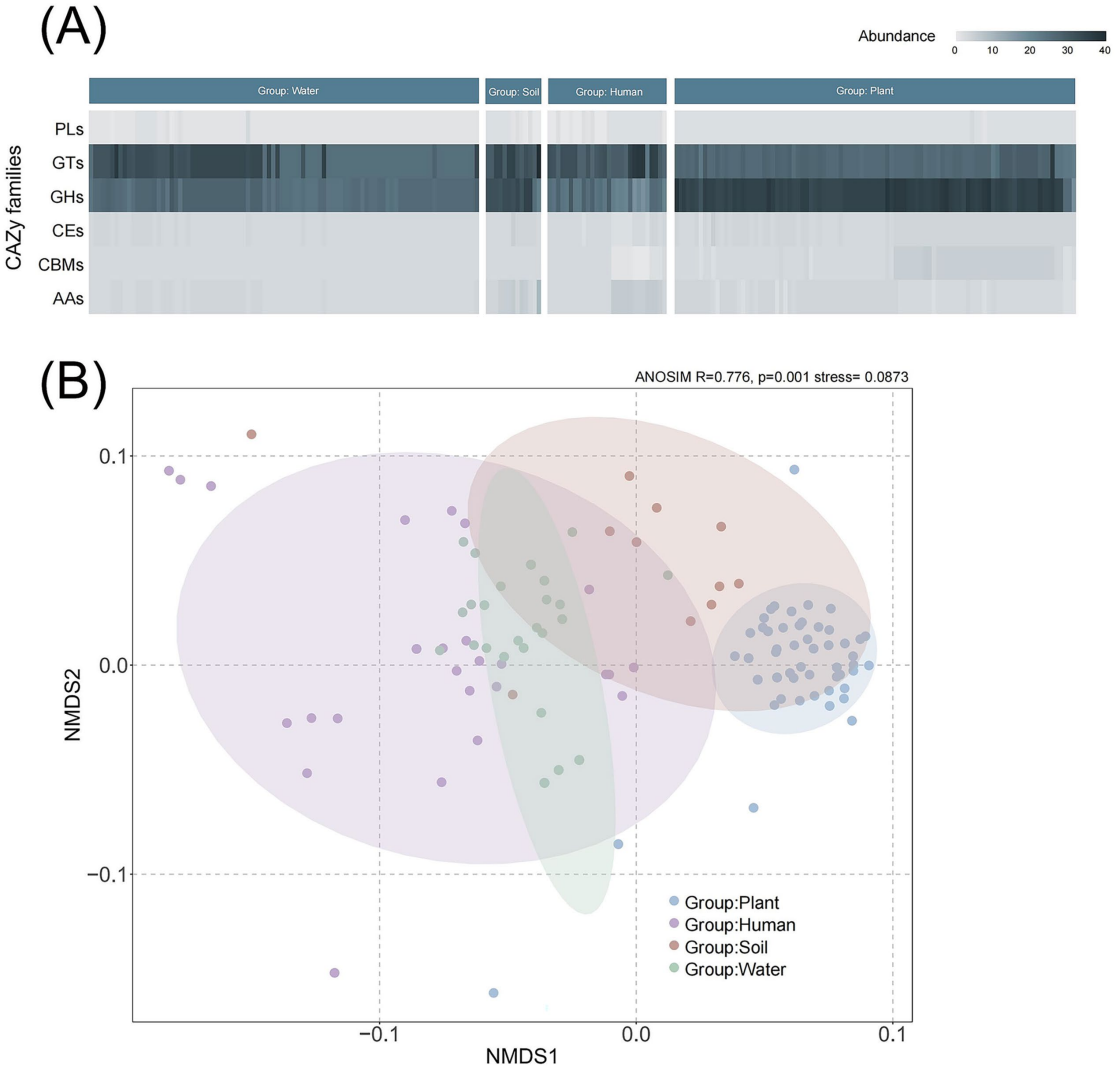
Composition and comparative analysis of carbohydrate-active enzymes (CAZymes) in *Ralstonia* spp. genomes. **(A)** Diversity and abundance of CAZymes across different clades. CAZyme genes are classified into six categories: PL (polysaccharide lyases), GT (glycosyltransferases), GH (glycoside hydrolases), CE (carbohydrate esterases), CBM (carbohydrate-binding modules), and AA (auxiliary activities). **(B)** NMDS (non-metric multidimensional scaling) analysis of enzyme composition among clades based on Bray–Curtis distance.

CAZymes are involved in biological processes related to carbohydrate synthesis and metabolism. They play roles in synthesis (GTs), degradation (GHs, PLs, CEs, AAs), and recognition (CBMs). The proportion of GHs, involved in carbohydrate degradation, was more abundant in the plant group compared to other groups (Figure 4A). Conversely, there were more GTs in the human group, which may be related to host invasion. There were no significant differences observed among PLs, CEs, CBMs, and AAs across the groups. The group of water species contains a different abundance GTs, indicating that different water habitat can also affect the composition of CAZymes. NMDS results revealed differences between the plant group and those groups with other habitats (R = 0.776, *p* = 0.001) based on bray-curtis distance (Figure 4B).

### 3.5 Metabolism analysis of *Ralstonia* spp.

**Figure 5:**
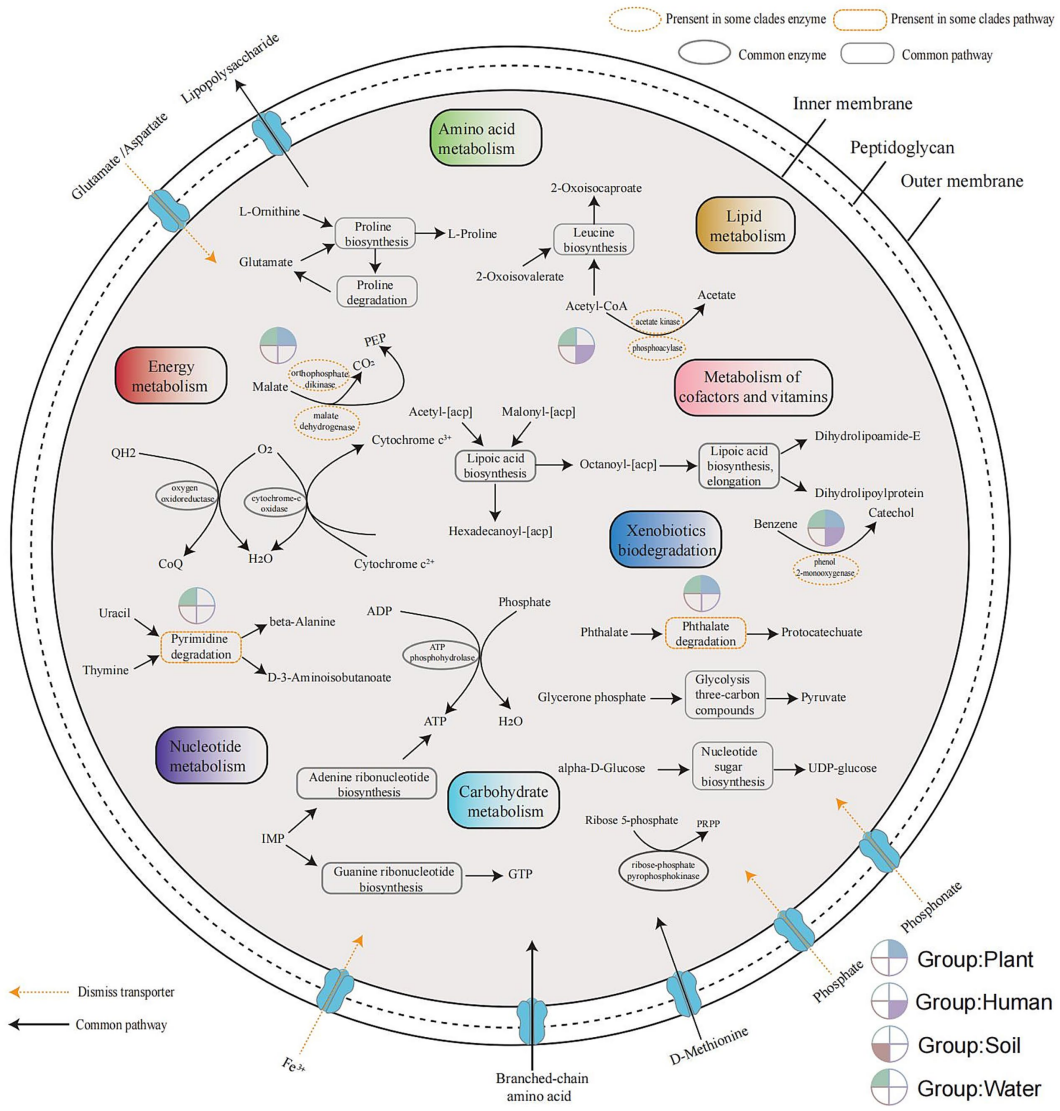
Overview of the metabolic potential across different habitats. Metabolic pathways were inferred from the orthogroup gene count matrix (excluding outgroups) obtained from OrthoFinder software results. Shared genomic metabolic pathways represent highly conserved sequences present in all genomes. Metabolic differences among clades were assessed using Fisher’s exact test (*p* < 0.05). Incomplete transporters, identified by sequence loss, were aligned to the corresponding *E. coli* protein sequences using BLASTp, with identity thresholds above 50%.

The metabolism of microorganisms is highly diverse. The core genome of the *Ralstonia* spp. consists of only 12 pathways or biochemical processes under strict conditions (present in all genomes), including amino acid metabolism (leucine biosynthesis, proline biosynthesis and degradation), energy metabolism (cytochrome-c oxidase and oxygen oxidoreductase), metabolism of cofactors and vitamins (lipoic acid biosynthesis), carbohydrate metabolism (glycolysis of three-carbon compounds, nucleotide sugar biosynthesis, ribose-phosphate pyrophosphokinase), and nucleotide metabolism (adenine ribonucleotide biosynthesis, guanine ribonucleotide biosynthesis, ATP phosphohydrolase). There are three shared ABC transporters, including absorption of branched-chain amino acids, D-methionine, and efflux of LPS (lipopolysaccharide). Dismiss transporters, defined as lacking one protein component, four inward transport systems as Fe^3+^, phosphate, phosphonate, and glutamate/aspartate, which were identified through whole-genome BLASTp searches (identity >50%) using *E. coli* sequences as reference indicated by the yellow dashed arrows in Figure 5.

The phthalate degradation and acetate synthesis pathways are observed in plant and water groups, while phenol 2-monooxygenase is absent in soil group. The pyrimidine degradation pathway is only present in the group of water, while PEP (phosphoenolpyruvate) synthesis is present in plant and water groups. There are four types of ABC transporters observed with dismiss based on BLASTp result in all genomes, including Fe^3+^, phosphate, phosphonate and glutamate/aspartate.

### 3.6 Secondary metabolite prediction from *Ralstonia* spp. genomes

**Figure 6:**
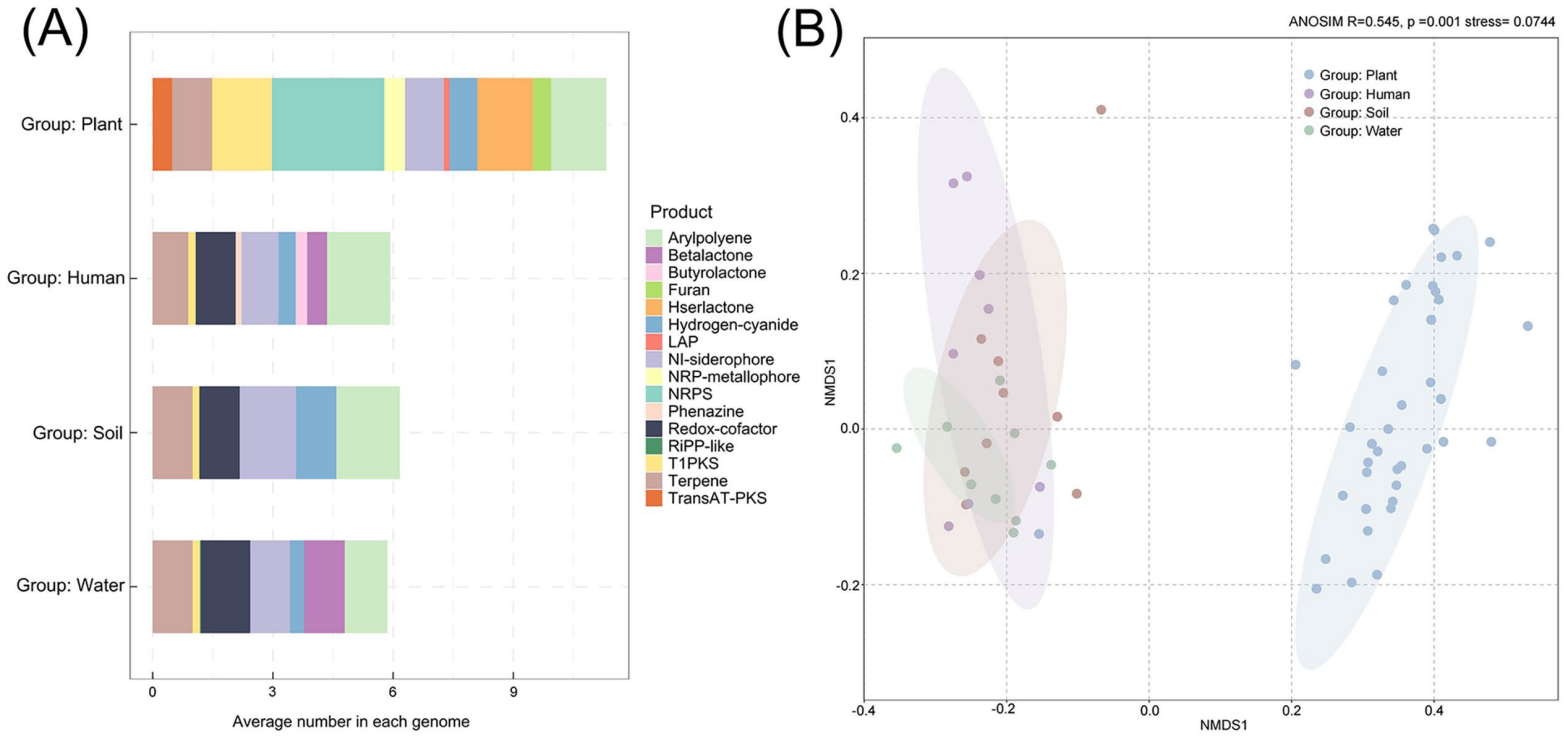
Prediction of secondary metabolites in *Ralstonia* spp. from different habitats based on antiSMASH software result. **(A)** Average number of secondary metabolites predicted per genome across different habitats. **(B)** NMDS analysis of clades based on secondary metabolite profiles using Bray–Curtis distances.

The prediction of secondary metabolites in *Ralstonia* spp. using antiSMASH software revealed a total of 16 secondary metabolites, with varying proportions observed across different habitats (Figure 6A). The average secondary metabolite content of each genome in different habitat is highest in the plant group, with NRPS (Nonribosomal peptides) content being the highest. However, in the human and soil groups, the average content of arylpolyene is the highest, while in water group, the content of redox-cofactor is the highest. Interestingly, the content of redox-cofactor in the plant group is extremely low, while the content of T1PKS is higher than the others. At the same time, the results of NMDS analysis also indicate differences in secondary metabolites between plant group and other groups (stress value <0.2, R = 0.545, *p* = 0.001) (Figure 6B).

## 4 Discussion

Our comparative genomic analysis sheds light on the ecological and evolutionary adaptations of *Ralstonia* spp., with a particular focus on the aquatic habitat lineage. By reconstructing the phylogenomic relationships of 228 genomes, we identified four clades based on dominate habitat (plant, human, soil, and water). Notably, the water-associated *R. pickettii* lineage exhibited unique genomic features, including reduced T3SS genes content, diverse ARGs, and a distinctive pyrimidine degradation pathway, all of which suggest adaptations to oligotrophic aquatic environments. These findings contribute to a deeper understanding of the ecological roles of *Ralstonia* spp., particularly in aquatic microbial communities.

### 4.1 The relationship between *Ralstonia pickettii* and *Dolichospermum* sp.

Algae bloom is an important manifestation of microecological disorder in the water environment, manifested by an abnormal increase in the abundance of algae, extremely serious in China (Huo et al., 2021). *Dolichospermum* as one of bloom-forming cyanobacterium can produce toxins, including microcystin, which inhibit protein phosphatases, enhance cell membrane permeability, and cause DNA damage (Ji et al., 2024; Hitzfeld et al., 2000; Jong et al., 2020; Winter et al., 2021). These effects heighten the selection pressure for ARGs and facilitate HGT, potentially leading to a greater diversity of ARGs in aquatic environments of *R. pickettii*. We isolate *R. pickettii* from cyanobacterium cultures, enabling to preliminarily explore its possible relationship with algae blooms. The connection appears to be initially related to EPS include proteins, glyoxylates and lipids, which is critical for microbial aggregation (Liu et al., 2023; Naveed et al., 2019; Guan et al., 2024) and the microbial community can produce VB_12_ to promote the growth of cyanobacteria (Xie et al., 2016). The FISH results (Figure 1) indicate that *R. pickettii* may free living in lakes and achieve coexistence with cyanobacteria in a free state in the culture. When *Dolichospermum* is under a low-nitrogen environment, *R. pickettii* can promote pyrimidine degradation, supplement nitrogen sources, and prevent *Dolichospermum* dying for nitrogen deficiency.

### 4.2 Inconsistencies between the phylogeny of certain species and ecological habitats

We performed a preliminary screening of genomes based on completeness and retrieved associated habitat information from the NCBI database at the time of genome submission. For genomes with incomplete or missing habitat annotations, additional information was obtained through manual curation from published literature and relevant databases. Phylogenetic analysis revealed four distinct clades, which we classified into four habitat-based groups: Plant, Human, Soil, and Water. These classifications were derived objectively from the available data, without any prior assumptions.

Notably, discrepancies were observed between the phylogenetic placement and the recorded habitats of certain species. This may be attributed to the high environmental adaptability of *Ralstonia* species. Previous studies have shown that strains of the *R. solanacearum* species complex (plant pathogens) can persist in water and soil for extended periods (Alvarez et al., 2008; de Pedro-Jové et al., 2023). Moreover, human activities such as water consumption and excretion may facilitate the introduction of human-associated strains into aquatic or soil environments, while surface runoff following rainfall may contribute to the transfer of soil-associated strains into water bodies.

### 4.3 T3SS deficiency and ARGs diversity increased in water group

Many *Ralstonia* species are opportunistic pathogens that infect humans and plants, causing respiratory failure and leading to significant economic losses, respectively (Fluit et al., 2021; Ryan et al., 2006). The reasons for the diversity of ARGs are closely related to the microenvironment, where ARGs can be transferred among various microorganisms through mobile genetic elements (MGEs), including transposons, plasmids, and insertion sequences, thereby facilitating the adaptive evolution of resistant bacteria in this environment (Bellanger et al., 2014; Gootz, 2010). ARGs may potentially interact with native microbes in freshwater and estuarine ecosystems, leading to modifications in bacterial ecology and subsequent changes in microbial community structure, which influence ecosystem sustainability and function (Ohore et al., 2022). Therefore, the increased diversity of ARGs (*OXA*, *sul2* and *ceoB*) in the aquatic community observed in this study may linked to the water ecological environment.

Gram-negative bacteria possess six types of secretion systems, labeled from I to VI. *R. solanacearum* relies on the TSS for the production of EPS, cell appendages, and protein secretions (Costa et al., 2015). In certain microbial communities, Bacteroidales utilize the T6SS system to shape the formation and evolution of these communities (García-Bayona et al., 2021). Simultaneously, the triggered of T4SS also facilitates the dissemination of resistance genes within microbial communities (Zhao et al., 2021). The absence of T3SS in certain species found in the human host group may be attributable to their origin in aquatic environments preceding human colonization. The absence of T3SS-associated genes in aquatic *Ralstonia* spp., in contrast to their terrestrial counterparts, suggests a distinct adaptive strategy to life in aquatic environments.

### 4.4 CAZymes and secondary metabolites different between plant group and others

Additionally, we explored the survival patterns of different groups in the habitats through CAZymes differences. CAZymes exhibit a higher prevalence in the genomes of pathogens, potentially associated with the degradation of complex carbohydrate structures within hosts, such as plant cell walls (Huang et al., 2018). Currently, GTs have been implicated in various pathogenic bacteria, including enterotoxigenic *E. coli*, *Photorhabdus asymbiotica*, *Pseudomonas aeruginosa* (Szymanski and Wren, 2005; Lu et al., 2015). In this study, the abundance of GTs showed variation even in the water group, potentially due to differences in aquatic environments. The increased synthesis of polysaccharides might contribute to the enhanced adaptability of bacteria to complex aquatic environment.

Secondary metabolites are substances that use primary metabolites as precursors and do not have specific biological functions. Due to differences in hosts or environments, there are significant distinctions between plant-associated groups and other groups. For example, NRPS, a diverse class of peptide compounds, plays an important role as an antibiotic in clinical applications, including telomycin, griselimycin, and lugdunin (Dang and Süssmuth, 2017). These findings suggest that plant-associated *Ralstonia* spp. possess distinct adaptive traits that may contribute to their successful colonization and survival within specific ecological niches. The proportion of redox-cofactor and betalactone are the highest in aquatic environments than other habitats. Redox cofactor are molecules that contains multiple types and can enhance the ability of microorganisms to adapt to various extreme environments (Somayaji et al., 2022). A redox cofactor enables microorganisms to adapt to shifts in redox states across diverse and complex environments, potentially associate its function with the variability of aquatic ecosystems (Guedes da Silva et al., 2020). Betalactone compounds contain multiple antibiotics to enhance microbial resistance to bacterial and fungal invasions and are an important way to obtain dominant community ecological niches (Tymiak et al., 1985; Robinson et al., 2019). The results indicate that *Ralstonia* spp. has adapted to a variable environment and produced different types of secondary metabolites.

### 4.5 A distinctive pyrimidine degradation pathway in the water group

The metabolism of microorganisms is diverse, consists of only 12 pathways or biochemical processes represents an extremely conservative function under strict conditions (present in all genomes). Although many metabolic pathways in the phylum Proteobacteria are known to be conserved, our predictions were based on protein annotations derived from genome data using computational tools. Due to inherent limitations in reference databases and algorithmic approaches, these predictions may be incomplete. However, the probability of incomplete annotation is expected to be consistent across all genomes. Therefore, instead of focusing solely on conserved core metabolic functions, our primary interest lies in identifying adaptive metabolic differences associated with distinct environmental niches. In the water group, the metabolic types are the most abundant, the group has acetate metabolism, phenol 2-monooxygenase, phthalates, malate dehydrogenase and PEP production. The pyrimidine degradation pathway enables assimilation of nitrogen and carbon for growth (Yin et al., 2019). *R. pickettii* in the water group employs this pyrimidine degradation strategy, which may relate to its ability to survive in relatively oligotrophic media (Matache et al., 2024). There is an abundance of pyrimidine in water, and its degradation produces urea. This urea serves as a primary nitrogen source for microorganisms, enabling them to adapt to low-nitrogen environments such as the space environment (Berg and Jørgensen, 2006; Maruyama et al., 2009; Santiago and West, 1999). Moreover, increased urea production may enhance nitrogen fixation by *Dolichospermum* sp., suggesting a potential interaction between *R. pickettii* and *Dolichospermum* sp.

In conclusion, this study highlights the ecological and evolutionary adaptations of *Ralstonia* spp., revealing four clades associated with soil, water, plant, and human groups based on dominate habitats. Water-associated *R. pickettii* strains show unique adaptations to aquatic environments, including reduced T3SS gene content, and a pyrimidine degradation pathway that supports survival in oligotrophic conditions. ARGs such as *OXA* and *ceoB*, enriched in water-associated strains, underscore the dynamic nature of microbial communities in aquatic ecosystems. Secondary metabolite profiles further reveal habitat-specific metabolic strategies. In water-associated strains, redox cofactors are likely to enhance resilience to oxidative stress, while *β*-lactones may confer competitive advantages. During algal bloom events, *R. pickettii* appears to coexist with cyanobacteria, potentially utilizing extracellular polymeric substances as a nutrient source. These findings provide new insights into the adaptive evolution of *Ralstonia* spp., particularly in aquatic ecosystems, and raise important questions about the ecological roles of *R. pickettii* during cyanobacterial blooms.

## 5 Limitations of the study

Due to the fact that this study is based on genome prediction results, additional direct experimental evidence is needed to support this interaction. Establish an indoor simulation group to observe the growth of *Dolichospermum* sp. and *R. pickettii* in coexistence, as well as the response to adding *R. pickettii* at different EPS concentrations. However, due to the difficulty in purifying *Dolichospermum*, indoor simulation experiments continue to encounter significant challenges. Additionally, we sought to verify whether the ability to degrade pyrimidine is exclusive to these groups. However, this idea has proven difficult to pursue due to the lack of available strains of different habitats.

## Data Availability

The original contributions presented in the study are publicly available. This data can be found here: https://www.ncbi.nlm.nih.gov/genbank/, PRJNA1111332.

## Footnotes

^1^ http://blast.ncbi.nlm.nih.gov/Blast.cgi

^2^ https://github.com/kblin/ncbi-genome-download

^3^ https://github.com/tseemann/barrnap

^4^ http://blast.ncbi.nlm.nih.gov/Blast.cgi

^5^ https://github.com/tseemann/abricate

^6^ https://antismash.secondarymetabolites.org/#!/start

^7^ https://www.kegg.jp/kegg/mapper/reconstruct.html
