# Supplementary figures and images for: Comparative genomic analysis reveals the adaptive traits of Ralstonia spp. in aquatic environments
Source: Front Microbiol. 2025 Jul 30;16:1625651. doi: 10.3389/fmicb.2025.1625651 (PMC12345375; doi:10.3389/fmicb.2025.1625651)

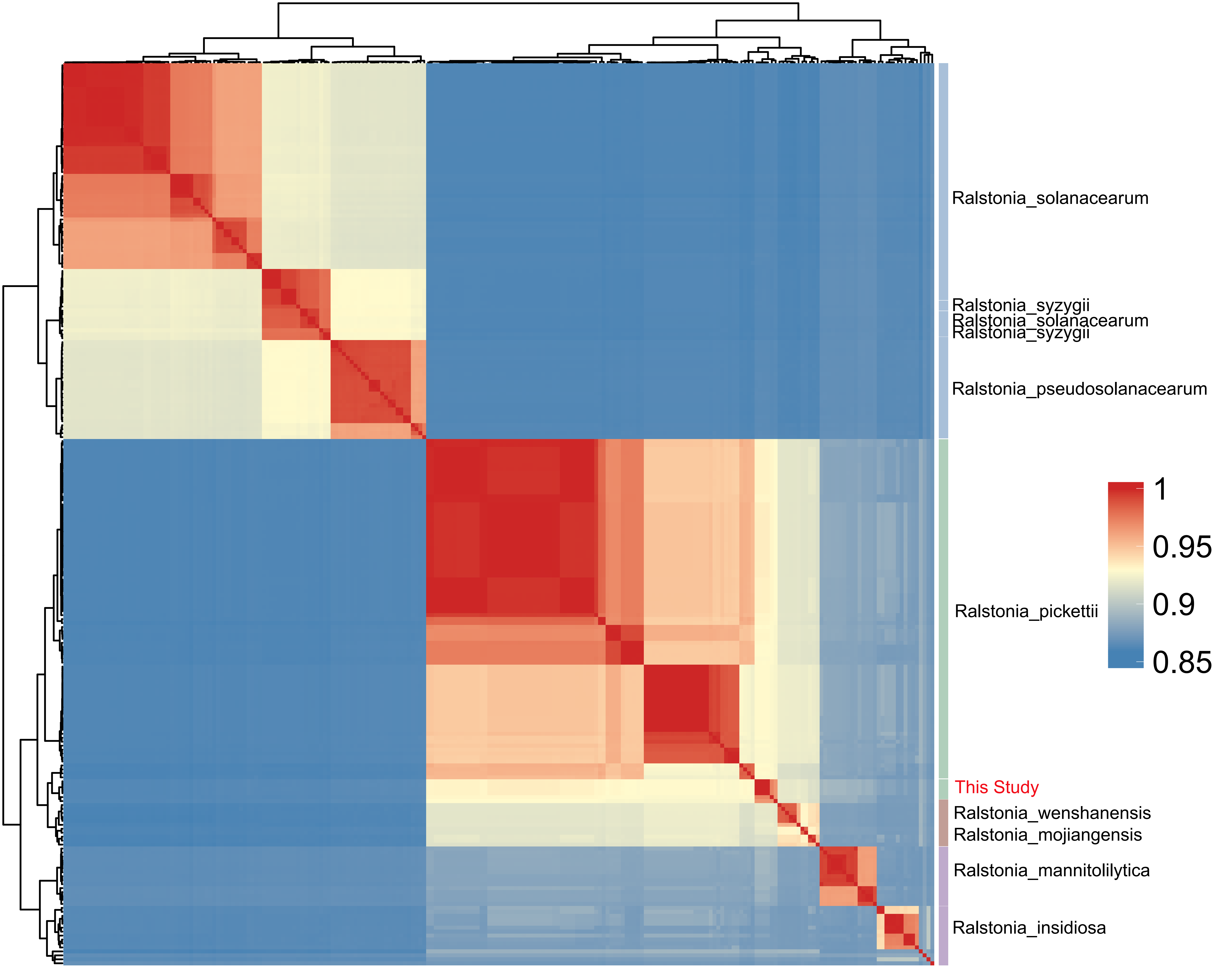

Supplement: Supplementary file 1 [file Image_1.PNG]

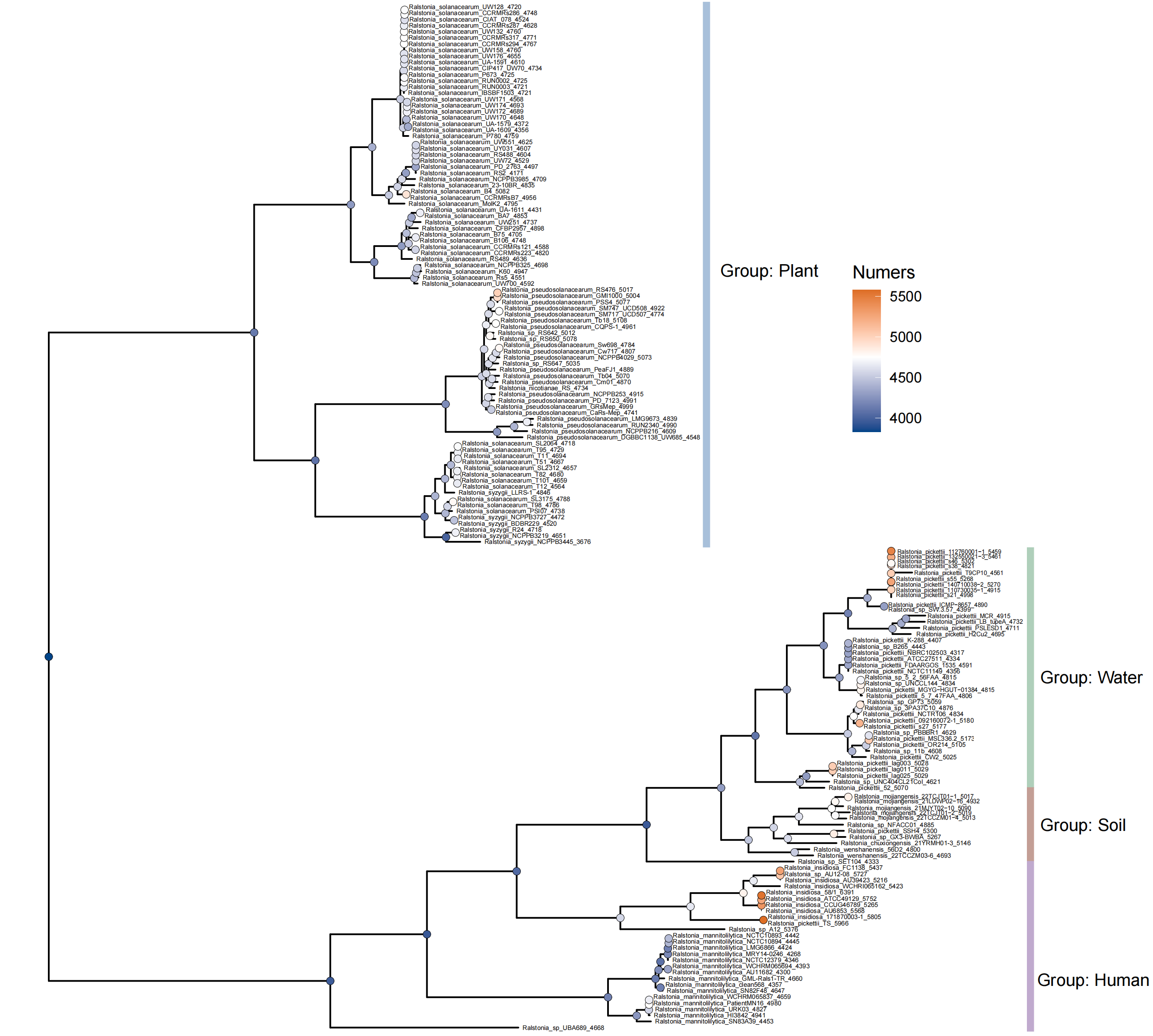

Supplement: Supplementary file 2 [file Image_2.PNG]
