# Supplementary material for: Comparative genomic analysis reveals the adaptive traits of Ralstonia spp. in aquatic environments
Source: Front Microbiol. 2025 Jul 30;16:1625651. doi: 10.3389/fmicb.2025.1625651 (PMC12345375; doi:10.3389/fmicb.2025.1625651)
Supplement: Supplementary file 3 [file Supplementary_file_1.DOCX]

**Supplementary Material**

**Comparative genomic analysis reveals the adaptive traits of Ralstonia spp. in aquatic environments**

Gaopeng Liu, Chengzhi Mao, Qi Li, Da Huo and Tao Li

**Supplementary results**

**Table S1**

| Name | Genome_size(bp) | contig_numbers | CDS(bp) | G+C (%) | RefSeq genome accession |
| --- | --- | --- | --- | --- | --- |
| *Ralstonia solanacearum* P780 | 5512323 | 97 | 4879 | 66.66 | GCF_023646535.1 |
| *Ralstonia pseudosolanacearum* SM747_UCD508 | 5703275 | 96 | 5058 | 67.04 | GCF_030518885.1 |
| *Ralstonia solanacearum* 23-10BR | 5629362 | 94 | 5037 | 66.66 | GCF_000749995.1 |
| *Ralstonia pseudosolanacearum* Tb04 | 5896040 | 93 | 5281 | 66.88 | GCF_023130705.1 |
| *Ralstonia solanacearum* UW190 | 5492905 | 93 | 4891 | 66.59 | GCF_028464665.1 |
| *Ralstonia pickettii* s1 | 5614868 | 91 | 5404 | 63.71 | GCF_014142725.1 |
| *Ralstonia solanacearum* UW176 | 5365472 | 89 | 4772 | 66.72 | GCF_028464775.1 |
| *Ralstonia solanacearum* UW158 | 5457915 | 87 | 4889 | 66.6 | GCF_028464865.1 |
| *Ralstonia pseudosolanacearum* DGBBC1138_UW685 | 5406075 | 85 | 4755 | 67.01 | GCF_023075375.1 |
| *Ralstonia solanacearum* UW128 | 5494760 | 83 | 4878 | 66.59 | GCF_023646455.1 |
| *Ralstonia solanacearum* UW132 | 5481571 | 83 | 4881 | 66.57 | GCF_028464965.1 |
| *Ralstonia pseudosolanacearum* SM717_UCD507 | 5594681 | 83 | 4896 | 67.09 | GCF_030518935.1 |
| *Ralstonia solanacearum* CCRMRs286 | 5467440 | 81 | 4853 | 66.61 | GCF_028177125.1 |
| *Ralstonia solanacearum* CCRMRs294 | 5477764 | 81 | 4872 | 66.6 | GCF_028177135.1 |
| *Ralstonia solanacearum* CCRMRs283 | 5467851 | 81 | 4854 | 66.61 | GCF_028177305.1 |
| *Ralstonia solanacearum* UW177 | 5366744 | 81 | 4765 | 66.72 | GCF_028464765.1 |
| *Ralstonia pickettii* 153490002-2 | 5818597 | 80 | 5542 | 63.31 | GCF_019641815.1 |
| *Ralstonia pickettii* 112620001-1 | 5819024 | 80 | 5539 | 63.31 | GCF_019642795.1 |
| *Ralstonia solanacearum* CCRMRs317 | 5499868 | 80 | 4896 | 66.57 | GCF_028177545.1 |
| *Ralstonia pickettii* 132550021-3 | 5818044 | 79 | 5538 | 63.31 | GCF_019642335.1 |
| *Ralstonia* sp. AU12-08 | 6229152 | 78 | 5815 | 63.53 | GCF_000442475.1 |
| *Ralstonia pickettii* 112060001-1 | 5818822 | 78 | 5537 | 63.31 | GCF_019642855.1 |
| *Ralstonia pickettii* 112760001-1 | 5819405 | 77 | 5538 | 63.31 | GCF_019642775.1 |
| *Ralstonia solanacearum* B75 | 5424833 | 77 | 4805 | 66.68 | GCF_028177505.1 |
| *Ralstonia pickettii* 151470044-2 | 5819415 | 75 | 5538 | 63.31 | GCF_019641895.1 |
| *Ralstonia pickettii* s64 | 5815661 | 74 | 5536 | 63.31 | GCF_014141585.1 |
| *Ralstonia pickettii* 153490002-3 | 5818764 | 74 | 5541 | 63.31 | GCF_019641775.1 |
| *Ralstonia pseudosolanacearum* Cm01 | 5720814 | 74 | 5039 | 67.02 | GCF_023130835.1 |
| *Ralstonia pickettii* s48 | 5815483 | 73 | 5537 | 63.31 | GCF_014141825.1 |
| *Ralstonia pickettii* ICMP-8657 | 5484397 | 72 | 4992 | 63.55 | GCF_002516395.1 |
| *Ralstonia pickettii* 133170061-1 | 5815339 | 72 | 5532 | 63.31 | GCF_019642455.1 |
| *Ralstonia pickettii* 113330051-2 | 5818912 | 72 | 5538 | 63.31 | GCF_019642735.1 |
| *Ralstonia pseudosolanacearum* Tb18 | 5942602 | 72 | 5314 | 66.86 | GCF_023130655.1 |
| *Ralstonia pickettii* s32 | 5816172 | 70 | 5537 | 63.31 | GCF_014142135.1 |
| *Ralstonia pickettii* s30 | 5814334 | 70 | 5529 | 63.31 | GCF_014142165.1 |
| *Ralstonia pickettii* s65 | 5815728 | 69 | 5537 | 63.31 | GCF_014141555.1 |
| *Ralstonia pickettii* s29 | 5814031 | 69 | 5532 | 63.31 | GCF_014142195.1 |
| *Ralstonia insidiosa* TS | 6468200 | 69 | 6126 | 63.31 | GCF_023217975.1 |
| *Ralstonia solanacearum* P673 | 5549250 | 69 | 4888 | 66.66 | GCF_023646435.1 |
| *Ralstonia solanacearum* RUN0002 | 5549250 | 69 | 4888 | 66.66 | GCF_023646475.1 |
| *Ralstonia solanacearum* RUN0003 | 5548179 | 69 | 4887 | 66.65 | GCF_023646515.1 |
| *Ralstonia pickettii* s49 | 5813208 | 68 | 5532 | 63.31 | GCF_014141745.1 |
| *Ralstonia pickettii* s34 | 5815866 | 68 | 5535 | 63.31 | GCF_014142125.1 |
| *Ralstonia mannitolilytica* WCHRM065694 | 4783043 | 67 | 4453 | 65.69 | GCF_002939115.1 |
| *Ralstonia pickettii* 110730035-1 | 5345646 | 67 | 4980 | 63.57 | GCF_019643075.1 |
| *Ralstonia* sp. 3PA37C10 | 5303655 | 65 | 4991 | 63.74 | GCF_005503495.1 |
| *Ralstonia pickettii* 140710038-2 | 5700113 | 65 | 5343 | 63.45 | GCF_019642175.1 |
| *Ralstonia pickettii* s19 | 5342851 | 63 | 4978 | 63.57 | GCF_014142405.1 |
| *Ralstonia pickettii* 092570011-1 | 5783836 | 63 | 5442 | 63.42 | GCF_019642025.1 |
| *Ralstonia pickettii* 092570008-1 | 5782962 | 63 | 5443 | 63.42 | GCF_019642555.1 |
| *Ralstonia pickettii* 133170049-3 | 5698029 | 62 | 5338 | 63.45 | GCF_019642295.1 |
| *Ralstonia pickettii* s51 | 5696886 | 61 | 5338 | 63.45 | GCF_014141735.1 |
| *Ralstonia pickettii* s6 | 5782490 | 61 | 5440 | 63.42 | GCF_014142655.1 |
| *Ralstonia pickettii* 093490003-1 | 5344678 | 61 | 4977 | 63.57 | GCF_019641795.1 |
| *Ralstonia pickettii* 132550028-1 | 5700791 | 61 | 5378 | 63.32 | GCF_019642395.1 |
| *Ralstonia* sp. A12 | 6104714 | 60 | 5605 | 63.47 | GCF_000801955.1 |
| *Ralstonia pickettii* s4 | 5781367 | 60 | 5438 | 63.42 | GCF_014142685.1 |
| *Ralstonia pickettii* s55 | 5696810 | 59 | 5337 | 63.45 | GCF_014141695.1 |
| *Ralstonia pickettii* s50 | 5696434 | 59 | 5337 | 63.45 | GCF_014141805.1 |
| *Ralstonia pickettii* 092950004-1 | 5389165 | 59 | 5026 | 63.58 | GCF_019642275.1 |
| *Ralstonia* sp. SET104 | 4796748 | 58 | 4497 | 63.27 | GCF_003851545.1 |
| *Ralstonia pickettii* s7 | 5343083 | 58 | 4975 | 63.57 | GCF_014142645.1 |
| *Ralstonia* sp.SW.3.57 | 4797287 | 57 | 4457 | 63.9 | GCF_013824635.1 |
| *Ralstonia pickettii* s5 | 5386944 | 56 | 5022 | 63.58 | GCF_014142695.1 |
| *Ralstonia pickettii* 092160076-1 | 5430524 | 56 | 5063 | 63.56 | GCF_019642805.1 |
| *Ralstonia solanacearum* UW172 | 5388829 | 56 | 4780 | 66.67 | GCF_028464835.1 |
| *Ralstonia pickettii* NCTR106 | 5271161 | 55 | 5013 | 63.82 | GCF_018603815.1 |
| *Ralstonia pickettii* s46 | 5698483 | 54 | 5375 | 63.32 | GCF_014141845.1 |
| *Ralstonia pickettii* s3 | 5428919 | 54 | 5061 | 63.56 | GCF_014142735.1 |
| *Ralstonia pickettii* 110730038-1 | 5431051 | 54 | 5062 | 63.56 | GCF_019643015.1 |
| *Ralstonia pickettii* 093350054-1 | 5431053 | 53 | 5062 | 63.56 | GCF_019641695.1 |
| *Ralstonia solanacearum* CCRMRs223 | 5578840 | 53 | 4926 | 66.63 | GCF_028177285.1 |
| *Ralstonia solanacearum* UW174 | 5385000 | 53 | 4786 | 66.69 | GCF_028464785.1 |
| *Ralstonia pickettii* NBRC 102503 | 4733058 | 52 | 4377 | 63.97 | GCF_001544155.1 |
| *Ralstonia pickettii* s8 | 5430218 | 52 | 5061 | 63.56 | GCF_014142625.1 |
| *Ralstonia pickettii* s21 | 5429456 | 51 | 5059 | 63.56 | GCF_014142365.1 |
| *Ralstonia solanacearum* UW171 | 5268082 | 51 | 4651 | 66.8 | GCF_028464815.1 |
| *Ralstonia* sp. UNCCL144 | 5251611 | 51 | 4923 | 63.63 | GCF_900099845.1 |
| *Ralstonia pickettii* 121850007-2 | 5257754 | 50 | 4896 | 63.66 | GCF_019642675.1 |
| *Ralstonia solanacearum* B4 | 5858492 | 50 | 5169 | 66.48 | GCF_028177185.1 |
| Ralstonia mannitolilytica MRY14-0246 | 4669233 | 48 | 4348 | 65.78 | GCF_000953875.1 |
| *Ralstonia* sp. UBA689 | 5216173 | 48 | 4859 | 64.15 | GCF_002298975.1 |
| *Ralstonia pickettii* T9CP10 | 4985044 | 48 | 4706 | 63.66 | GCF_013413505.1 |
| *Ralstonia pickettii* s38 | 5256159 | 47 | 4893 | 63.66 | GCF_014142025.1 |
| *Ralstonia pickettii* OR214 | 5480587 | 46 | 5215 | 63.41 | GCF_000372665.1 |
| *Ralstonia insidiosa* WCHRI065162 | 5924662 | 46 | 5558 | 63.41 | GCF_002939165.1 |
| *Ralstonia pickettii* 123250053-1 | 5015890 | 46 | 4646 | 63.77 | GCF_019642515.1 |
| *Ralstonia solanacearum* B106 | 5497095 | 46 | 4848 | 66.62 | GCF_028177365.1 |
| *Ralstonia mannitolilytica* HI3842 | 5383646 | 45 | 5093 | 65 | GCF_019795175.1 |
| *Ralstonia pickettii* MSL336.2 | 5563662 | 44 | 5384 | 63.35 | GCF_023712645.1 |
| *Ralstonia* sp. 25MFCol4.1 | 6337400 | 44 | 5717 | 65.58 | GCF_900104095.1 |
| *Ralstonia pickettii* s43 | 5013749 | 43 | 4643 | 63.77 | GCF_014141915.1 |
| *Ralstonia pickettii* 110760020-1 | 5535017 | 43 | 5281 | 63.7 | GCF_019642915.1 |
| *Ralstonia pickettii* 101480035-1 | 5539325 | 42 | 5283 | 63.7 | GCF_019643215.1 |
| *Ralstonia pickettii* 092160072-1 | 5538813 | 41 | 5283 | 63.7 | GCF_019642995.1 |
| *Ralstonia insidiosa* 58/1 | 7029311 | 41 | 6542 | 63.57 | GCF_022214855.1 |
| *Ralstonia pickettii* H2Cu2 | 5200293 | 40 | 4805 | 63.99 | GCF_001699795.1 |
| *Ralstonia pickettii* s27 | 5534019 | 40 | 5278 | 63.7 | GCF_014142205.1 |
| *Ralstonia pickettii* s2 | 5538224 | 40 | 5279 | 63.7 | GCF_014142755.1 |
| *Ralstonia pickettii* 101480038-1 | 5534649 | 40 | 5278 | 63.7 | GCF_019643135.1 |
| *Ralstonia pickettii* 101120021-1 | 5536409 | 40 | 5281 | 63.7 | GCF_019643195.1 |
| *Ralstonia solanacearum* UW170 | 5412403 | 40 | 4787 | 66.66 | GCF_023646415.1 |
| *Ralstonia insidiosa* WCHRI065437 | 6115292 | 39 | 5727 | 63.63 | GCF_002939035.1 |
| *Ralstonia pickettii* s14 | 5534060 | 39 | 5276 | 63.7 | GCF_014142505.1 |
| *Ralstonia pickettii* s13 | 5537555 | 39 | 5279 | 63.7 | GCF_014142515.1 |
| *Ralstonia pickettii* s11 | 5535820 | 39 | 5279 | 63.7 | GCF_014142545.1 |
| *Ralstonia pickettii* 102690001-2 | 5352779 | 39 | 5065 | 63.76 | GCF_019643105.1 |
| *Ralstonia pickettii* SSH4 | 5746538 | 38 | 5375 | 63.29 | GCF_000607165.1 |
| *Ralstonia mannitolilytica* WCHRM065837 | 4990628 | 37 | 4715 | 65.79 | GCF_002939145.1 |
| *Ralstonia pickettii* s17 | 5351535 | 37 | 5064 | 63.76 | GCF_014142445.1 |
| *Ralstonia pickettii* 110700041-2 | 5494920 | 37 | 5221 | 63.72 | GCF_019642935.1 |
| *Ralstonia pickettii* 100540015-2 | 5348773 | 37 | 5059 | 63.77 | GCF_019643235.1 |
| *Ralstonia pickettii* s26 | 5494331 | 36 | 5218 | 63.72 | GCF_014142265.1 |
| *Ralstonia solanacearum* PD:2763 | 5263875 | 36 | 4644 | 66.65 | GCF_015912235.1 |
| *Ralstonia mannitolilytica* LMG 6866 | 4823626 | 36 | 4499 | 65.81 | GCF_905397375.1 |
| *Ralstonia pickettii* s10 | 5348194 | 35 | 5059 | 63.77 | GCF_014142575.1 |
| *Ralstonia solanacearum* CCRMRs121 | 5364378 | 35 | 4679 | 66.72 | GCF_028177115.1 |
| *Ralstonia* sp. GX3-BWBA | 5817683 | 34 | 5357 | 63.3 | GCF_003290055.1 |
| *Ralstonia pickettii* 5_7_47FAA | 5249816 | 32 | 4956 | 63.46 | GCF_000165085.1 |
| *Ralstonia pickettii* CW2 | 5490874 | 32 | 5147 | 63.65 | GCF_000607185.1 |
| *Ralstonia mannitolilytica* AU11682 | 4720405 | 31 | 4390 | 65.69 | GCF_019042415.1 |
| *Ralstonia soli* 21MJYT02-11 | 5726971 | 31 | 5261 | 64.12 | GCF_023955655.1 |
| *Ralstonia mannitolilytica* clean568 | 4740941 | 29 | 4431 | 65.83 | GCF_027319325.1 |
| *Ralstonia pickettii* PSLESD1 | 5267333 | 25 | 4871 | 63.82 | GCF_009668005.1 |
| *Ralstonia* sp. 11b | 5024091 | 25 | 4709 | 63.76 | GCF_031581505.1 |
| *Ralstonia* sp. NFACC01 | 5365746 | 25 | 4938 | 63.77 | GCF_900115545.1 |
| *Ralstonia* sp. GP73 | 5455776 | 23 | 5145 | 63.63 | GCF_029893465.1 |
| *Ralstonia* sp. PBBBR1 | 5034226 | 22 | 4726 | 63.82 | GCF_002256165.1 |
| *Ralstonia pseudosolanacearum* NCPPB 216 | 5430530 | 22 | 4910 | 66.63 | GCF_015910735.1 |
| *Ralstonia pseudosolanacearum* NCPPB 4029 | 6016317 | 20 | 5366 | 66.73 | GCF_015910515.1 |
| *Ralstonia insidiosa* AU39423 | 5685034 | 20 | 5270 | 63.65 | GCF_019795515.1 |
| *Ralstonia chuxiongensis* 21YRMH01-3 | 5612125 | 20 | 5226 | 63.47 | GCF_024158925.1 |
| *Ralstonia wenshanensis* 22TCCZM03-6 | 5167002 | 20 | 4744 | 63.91 | GCF_025290815.1 |
| *Ralstonia solanacearum* NCPPB 325 | 5652708 | 19 | 5002 | 66.43 | GCF_015910705.1 |
| *Ralstonia insidiosa* AU6853 | 6028148 | 19 | 5615 | 63.6 | GCF_019795305.1 |
| *Ralstonia syzygii* NCPPB 3219 | 5462846 | 18 | 5031 | 65.85 | GCF_015910645.1 |
| *Ralstonia mojiangensis* 22TCJT01-2 | 5615131 | 18 | 5086 | 63.5 | GCF_025290765.1 |
| *Ralstonia* sp. 5_2_56FAA | 5254771 | 17 | 4892 | 63.52 | GCF_000227255.2 |
| *Ralstonia pickettii* MGYG-HGUT-01384 | 5254771 | 17 | 4890 | 63.52 | GCF_902374465.1 |
| *Ralstonia syzygii* NCPPB 3445 | 4383940 | 16 | 4367 | 66.22 | GCF_015910655.1 |
| *Ralstonia mojiangensis* 22TCCZM01-4 | 5512593 | 16 | 5068 | 63.67 | GCF_025290755.1 |
| *Ralstonia insidiosa* CCUG 46789 | 5723831 | 15 | 5311 | 63.7 | GCF_008801405.1 |
| *Ralstonia mojiangensis* 21MJYT02-10 | 5603312 | 14 | 5166 | 63.55 | GCF_023955645.1 |
| *Ralstonia mojiangensis* 21LDWP02-16 | 5447773 | 14 | 4978 | 63.66 | GCF_025290735.1 |
| *Ralstonia solanacearum* CaRs-Mep | 5606203 | 13 | 4954 | 67 | GCF_001855495.2 |
| *Ralstonia pickettii* 52 | 5530053 | 13 | 5138 | 64.43 | GCF_002849525.1 |
| *Ralstonia solanacearum* NCPPB 3985 | 5610548 | 12 | 4869 | 66.75 | GCF_015910635.1 |
| *Ralstonia pseudosolanacearum* NCPPB 253 | 5798890 | 12 | 5112 | 66.94 | GCF_015910695.1 |
| *Ralstonia* sp. UNC404CL21Col | 5087452 | 11 | 4651 | 64.58 | GCF_000620465.1 |
| *Ralstonia solanacearum* UW700 | 5487557 | 11 | 4906 | 66.5 | GCF_002251605.2 |
| *Ralstonia pickettii* LB_tupeA | 5320276 | 11 | 4883 | 63.65 | GCF_013391365.1 |
| *Ralstonia pseudosolanacearum* PD:7123 | 5884479 | 11 | 5185 | 66.8 | GCF_015911755.1 |
| *Ralstonia pseudosolanacearum* GRsMep | 5837336 | 10 | 5130 | 66.95 | GCF_024460955.1 |
| *Ralstonia mannitolilytica* Patient MN16 | 5338286 | 10 | 5122 | 65.65 | GCF_030550865.1 |
| *Ralstonia mojiangensis* 22TCJT01-1 | 5560875 | 8 | 5076 | 63.54 | GCF_025290775.1 |
| *Ralstonia pickettii* FDAARGOS_1535 | 5008012 | 5 | 4664 | 63.8 | GCF_020341455.1 |
| *Ralstonia insidiosa* 171870003-1 | 6271672 | 5 | 5878 | 63.33 | GCF_028920815.1 |
| *Ralstonia pickettii* NCTC11149 | 4810167 | 5 | 4434 | 63.92 | GCF_900455835.1 |
| *Ralstonia solanacearum* UW163 | 5596238 | 4 | 4953 | 66.53 | GCF_001587135.1 |
| *Ralstonia insidiosa* ATCC 49129 | 6177004 | 4 | 5831 | 62.95 | GCF_001663855.1 |
| *Ralstonia mannitolilytica* GML-Rals1-TR | 5084397 | 4 | 4762 | 65.54 | GCF_002863525.1 |
| *Ralstonia syzygii* NCPPB 3727 | 5191832 | 4 | 4706 | 66.45 | GCF_015910595.1 |
| *Ralstonia mannitolilytica* URK03 | 5310746 | 4 | 5053 | 65.67 | GCF_031932125.1 |
| *Ralstonia mannitolilytica* SN82F48 | 5072301 | 3 | 4742 | 65.49 | GCF_000954135.1 |
| *Ralstonia insidiosa* FC1138 | 5987762 | 3 | 5573 | 63.64 | GCF_001653935.1 |
| *Ralstonia pickettii* K-288 | 4831420 | 3 | 4472 | 63.91 | GCF_016466415.2 |
| *Ralstonia syzygii* BDBR229 | 5231100 | 3 | 4744 | 66.42 | GCF_029219945.1 |
| *Ralstonia solanacearum* MolK2 | 5586409 | 3 | 4937 | 66.66 | GCF_029219985.1 |
| *Ralstonia solanacearum* BA7 | 5749371 | 3 | 5048 | 66.4 | GCF_029220005.1 |
| *Ralstonia solanacearum* CFBP2957 | 5692289 | 3 | 5021 | 66.43 | GCF_029220025.1 |
| *Ralstonia pickettii* MCR | 5356255 | 3 | 4992 | 63.75 | GCF_030582395.1 |
| *Ralstonia mannitolilytica* NCTC10894 | 4860652 | 3 | 4525 | 65.78 | GCF_900455575.1 |
| *Ralstonia pseudosolanacearum* GMI1000 | 5810922 | 2 | 5123 | 66.98 | GCF_000009125.1 |
| *Ralstonia solanacearum* Po82 | 5430263 | 2 | 4789 | 66.67 | GCF_000215325.1 |
| *Ralstonia solanacearum* PSI07 | 5605618 | 2 | 4882 | 66.32 | GCF_000283475.1 |
| *Ralstonia solanacearum* UY031 | 5411683 | 2 | 4770 | 66.61 | GCF_001299555.1 |
| *Ralstonia solanacearum* KACC 10722 | 5463256 | 2 | 4837 | 66.43 | GCF_001586135.1 |
| *Ralstonia solanacearum* IBSBF1503 | 5530249 | 2 | 4830 | 66.64 | GCF_001587155.1 |
| *Ralstonia mannitolilytica* SN83A39 | 4842994 | 2 | 4552 | 66.04 | GCF_001628775.1 |
| *Ralstonia pseudosolanacearum* CQPS-1 | 5893444 | 2 | 5230 | 66.84 | GCF_002220465.1 |
| *Ralstonia solanacearum* UW551 | 5478976 | 2 | 4808 | 66.55 | GCF_002251655.1 |
| *Ralstonia solanacearum* K60 | 5770663 | 2 | 5162 | 66.39 | GCF_002251695.1 |
| *Ralstonia solanacearum* RS 488 | 5411685 | 2 | 4763 | 66.55 | GCF_002501565.1 |
| *Ralstonia solanacearum* RS 489 | 5411504 | 2 | 4748 | 66.54 | GCF_002549815.1 |
| *Ralstonia solanacearum* T51 | 5400849 | 2 | 4775 | 66.43 | GCF_003515145.1 |
| *Ralstonia solanacearum* T11 | 5450627 | 2 | 4821 | 66.35 | GCF_003515165.1 |
| *Ralstonia solanacearum* SL3175 | 5555993 | 2 | 4894 | 66.36 | GCF_003515185.1 |
| *Ralstonia solanacearum* T98 | 5555978 | 2 | 4897 | 66.36 | GCF_003515265.1 |
| *Ralstonia solanacearum* T12 | 5520985 | 2 | 4864 | 66.42 | GCF_003515325.1 |
| *Ralstonia solanacearum* SL2312 | 5521456 | 2 | 4794 | 66.42 | GCF_003515425.1 |
| *Ralstonia solanacearum* SL2064 | 5473607 | 2 | 4840 | 66.41 | GCF_003515445.1 |
| *Ralstonia solanacearum* T101 | 5521368 | 2 | 4811 | 66.42 | GCF_003515485.1 |
| *Ralstonia solanacearum* T95 | 5474514 | 2 | 4839 | 66.41 | GCF_003515505.1 |
| *Ralstonia solanacearum* T82 | 5521457 | 2 | 4801 | 66.42 | GCF_003515525.1 |
| *Ralstonia pseudosolanacearum* RS476 | 5810927 | 2 | 5123 | 66.97 | GCF_003595305.1 |
| *Ralstonia solanacearum* CIAT_078 | 5389324 | 2 | 4853 | 66.69 | GCF_012562465.1 |
| *Ralstonia solanacearum* CCRMRsB7 | 5849612 | 2 | 5040 | 64.06 | GCF_014210345.1 |
| *Ralstonia solanacearum* CCRMRs287 | 5423031 | 2 | 4743 | 65.71 | GCF_014210375.1 |
| *Ralstonia solanacearum* Rs5 | 5430180 | 2 | 4778 | 66.56 | GCF_014884745.1 |
| *Ralstonia syzygii* LLRS-1 | 5694719 | 2 | 4998 | 66.31 | GCF_018243215.1 |
| *Ralstonia nicotianae* RS | 5613239 | 2 | 4861 | 67.1 | GCF_018243235.1 |
| *Ralstonia* sp. B265 | 4939245 | 2 | 4564 | 63.94 | GCF_018726805.1 |
| *Ralstonia solanacearum* UW72 | 5331181 | 2 | 4674 | 66.65 | GCF_021117095.1 |
| *Ralstonia solanacearum* CIP417_UW70 | 5485324 | 2 | 4843 | 66.67 | GCF_021117115.1 |
| *Ralstonia solanacearum* UW251 | 5572607 | 2 | 4838 | 66.63 | GCF_021117135.1 |
| *Ralstonia wenshanensis* 56D2 | 5310536 | 2 | 4856 | 63.74 | GCF_021173085.1 |
| *Ralstonia pseudosolanacearum* PeaFJ1 | 5808912 | 2 | 5058 | 66.85 | GCF_023518395.1 |
| *Ralstonia pseudosolanacearum* LMG 9673 | 5690309 | 2 | 5048 | 66.61 | GCF_024925465.1 |
| *Ralstonia pseudosolanacearum* Cw717 | 5592449 | 2 | 4948 | 67.03 | GCF_025859515.1 |
| *Ralstonia pseudosolanacearum* Sw698 | 5562184 | 2 | 4921 | 67.03 | GCF_025859635.1 |
| *Ralstonia* sp. RS642 | 5838575 | 2 | 5191 | 67 | GCF_026016705.1 |
| *Ralstonia* sp. RS650 | 5915133 | 2 | 5230 | 66.91 | GCF_026016725.1 |
| *Ralstonia* sp. RS647 | 5916874 | 2 | 5241 | 66.86 | GCF_026016745.1 |
| *Ralstonia syzygii* R24 | 5459644 | 2 | 4963 | 65.86 | GCF_029219965.1 |
| *Ralstonia pseudosolanacearum* RUN2340 | 5761046 | 2 | 5178 | 66.58 | GCF_029220045.1 |
| *Ralstonia pseudosolanacearum* PSS4 | 5879613 | 2 | 5183 | 66.92 | GCF_029220065.1 |
| *Ralstonia mannitolilytica* NCTC10893 | 4858077 | 2 | 4522 | 65.78 | GCF_900455685.1 |
| *Ralstonia mannitolilytica* NCTC12379 | 4752409 | 2 | 4423 | 65.88 | GCF_900461715.1 |
| *Ralstonia pickettii* lag003 | 5484666 | 2 | 5030 | 64.21 | lag003.fna |
| *Ralstonia pickettii* lag011 | 5484665 | 2 | 5031 | 64.21 | lag011.fna |
| *Ralstonia pickettii* lag025 | 5484672 | 2 | 5030 | 64.21 | lag025.fna |
| *Ralstonia pickettii* lag026 | 5501510 | 2 | 5045 | 64.21 | lag026.fna |
| *Ralstonia pickettii* ATCC 27511 | 4765599 | 1 | 4414 | 63.92 | GCF_000743455.1 |
| *Ralstonia solanacearum* RS2 | 4788265 | 1 | 4277 | 66.73 | GCF_001373295.1 |
| *Ralstonia solanacearum* UA-1611 | 5195693 | 1 | 4673 | 66.8 | GCF_003860685.1 |
| *Ralstonia solanacearum* UA-1591 | 5351985 | 1 | 4761 | 66.72 | GCF_003860705.1 |
| *Ralstonia solanacearum* UA-1579 | 5081426 | 1 | 4542 | 67.01 | GCF_003860725.1 |
| *Ralstonia solanacearum* UA-1617 | 5362479 | 1 | 4767 | 66.71 | GCF_003860745.1 |
| *Ralstonia solanacearum* UA-1609 | 5068052 | 1 | 4325 | 67.02 | GCF_003860765.1 |
| *Burkholderia cenocepacia* MSMB384WGS | 7780598 | 3 | 7016 | 67.25 | GCF_001718895.1 |
| *Burkholderia oklahomensis* C6786 | 7135022 | 2 | 6301 | 67.07 | GCF_000959365.1 |
| *Burkholderia plantarii* ATCC43733 | 8081051 | 3 | 6982 | 68.55 | GCF_000959365.1 |

**Fig. S1 Legend**

Fig. S1 Analysis of average nucleotide identity in a total of 228 *Ralstonia* genus genomes. Pyani (v 0.2.12) was used to calculate the average nucleotide identity (ANI) for further species identification based on ANIm algorithm. The depth of color represents the size of similarity among genomes.

**Fig. S2 Legend**

Fig. S2 Analysis of gene family contraction and expansion in *Ralstonia* genus used Badirate software based on BDI-CSP-FR model, which has removed duplicate values using ML tree. Blue represents plant-host associate habitat, green represents water habitat, brown represents soil habitat, and purple represents human-host associate habitat. Color blocks serve as a representation of quantity, with darker hues indicating a higher number of genes.
